# Risk factors for hepatocellular carcinoma in treated chronic hepatitis C patients–Relationship to smoking and alcohol

**DOI:** 10.1002/jgh3.12331

**Published:** 2020-04-16

**Authors:** Tomoka Matsuura, Satoko Ohfuji, Masaru Enomoto, Akihiro Tamori, Shoji Kubo, Kiyohide Kioka, Norifumi Kawada, Wakaba Fukushima

**Affiliations:** ^1^ Department of Public Health Osaka City University Graduate School of Medicine Osaka Japan; ^2^ Department of Hepatology Osaka City University Graduate School of Medicine Osaka Japan; ^3^ Department of Hepato‐Biliary‐Pancreatic Surgery Osaka City University Graduate School of Medicine Osaka Japan; ^4^ Department of Hepatology Osaka City General Hospital Osaka Japan

**Keywords:** alcohol consumption, hepatocellular carcinoma, risk factor, smoking, sustained virologic response

## Abstract

**Background and Aim:**

The purpose of this study was to identify lifestyle risk factors, such as cigarette smoking and alcohol consumption, in relation to the development of hepatocellular carcinoma (HCC) among chronic hepatitis C patients who have achieved a sustained virologic response (SVR).

**Methods:**

This cross‐sectional study was conducted between 2014 and 2017 using self‐administered questionnaires and medical information at two tertiary hospitals in Osaka, Japan. Study subjects were chronic hepatitis C patients who had achieved SVR without HCC following antiviral treatment that was completed more than 1 year earlier. A logistic regression model was used to calculate adjusted odds ratios (ORs) and 95% confidence intervals (CIs) for the development of post‐SVR HCC for each factor.

**Results:**

Of 202 participants, 18 patients were diagnosed with post‐SVR HCC. After considering potential confounders, former drinkers at the time of SVR (OR, 9.51; 95% CI, 1.08–83.90) and patients with a history of gastric or duodenal ulcer (OR, 4.14; 95% CI, 1.37–12.46) were significantly associated with HCC. In addition, among patients with severe fibrosis, current smokers at the time of SVR had an increased OR for HCC compared with never smokers, with marginal significance (OR, 5.61; 95% CI, 0.97–32.63).

**Conclusions:**

In chronic hepatitis C patients with severe fibrosis, continuing smoking after achieving SVR could be a risk factor for post‐SVR HCC. The relationship between gastric or duodenal ulcer history and post‐SVR HCC should be investigated further.

## Graphical abstract

A cross‐sectional study was conducted to investigate lifestyle risk factors associated with hepatocellular carcinoma (HCC) that developed in patients treated for chronic hepatitis C. Continued smoking might be a risk factor for HCC after sustained virilogical response (SVR), especially in patients with chronic hepatitis C with severe fibrosis. In addition, a history of gastric or duodenal ulcers could be a risk factor for developing post‐SVR HCC.

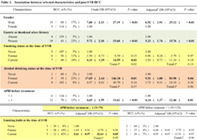

## Introduction

In recent years, remarkable advances in anti‐hepatitis C virus (HCV) therapeutics have enabled many chronic hepatitis C patients to achieve a sustained virologic response (SVR). Patients who achieved SVR were generally reported to have a lower incidence of hepatocellular carcinoma (HCC) compared to patients who did not achieve SVR, although some patients develop HCC even after achieving SVR (post‐SVR HCC).^1^, ^2^, ^3^, ^4^, ^5^, ^6^, ^7^, ^8^

The established risk factors for post‐SVR HCC after interferon (IFN) treatment are reportedly male gender, older age at the time of SVR, and severe fibrosis before treatment, as indicated by histological fibrosis grade F3 or F4, high aspartate aminotransferase (AST) level, low albumin level, and aspartate aminotransferase‐to‐platelet ratio index (APRI) ≥ 2.^1^, ^2^, ^3^, ^4^, ^5^ In addition, some articles reported high alpha‐fetoprotein (AFP) levels after treatment,^3^, ^4^ HCV genotype 3,^5^ and diabetes^5^ as risk factors for HCC. Regarding postdirect acting antiviral (DAA) treatment, two large studies reported similar associations as described above.^6^, ^7^, ^8^

In contrast, the effect of smoking and alcohol consumption on post‐SVR HCC remains to be elucidated as changes in these lifestyle habits might occur after achieving SVR due to symptoms of liver disease or under the doctor's guidance. Although smoking is a risk factor for the development of HCC in patients with chronic hepatitis C,^9^ the effect of smoking after achieving SVR on the risk of post‐SVR HCC has not been adequately examined. Regarding alcohol consumption, a history of heavy drinking was reported to be a risk factor for post‐SVR HCC.^5^, ^6^, ^7^, ^8^ However, the effect of light‐to‐moderate drinking on post‐SVR HCC has rarely been discussed. The aim of this study was to identify risk factors associated with post‐SVR HCC, focusing on smoking and drinking after achieving SVR.

## Methods

### *Study subjects*

This cross‐sectional study was conducted from September 2014 to March 2017 at the Departments of Hepatology and of Hepato‐Biliary‐Pancreatic Surgery at Osaka City University Hospital and the Department of Hepatology at Osaka City General Hospital, both of which are tertiary hospitals in Japan. The scale of the hospitals is almost the same, and socioeconomic status in each catchment area is similar. Eligible patients were outpatients at the cooperating hospitals who achieved SVR after anti‐HCV treatment and had completed the treatment more than 1 year earlier. Exclusion criteria were histories of liver cancer before achieving SVR, other hepatic comorbidities such as chronic hepatitis B or autoimmune hepatitis, HCV genotype 3, and diagnosis of HCC within 1 year after achieving SVR. The study protocol was approved by the ethics committee of Osaka City University Graduate School of Medicine (No. 3898; Osaka, Japan), and it was performed in accordance with the Declaration of Helsinki. Each attending physician explained the contents of the study verbally and provided a self‐administered questionnaire to eligible patients. Participants' consent was implied by the return of the questionnaires.

### *Data collection*

At the time of recruitment, each subject filled out a self‐administered questionnaire that included the following items: demographic and anthropometric characteristics (gender, age, height, weight); history of HCV treatment, including age and duration of treatment, blood transfusion history, medical history based on physician's diagnosis, and patient's age at diagnosis (e.g. diabetes, hypertension, hyperlipidemia, gastric or duodenal ulcer); and lifestyle habits (e.g. smoking, alcohol consumption). Subjects were asked about smoking status at the time of recruitment (never, former, or current), age when they started smoking, and daily smoking amount (cigarettes/day). Former smokers were additionally asked about age at quitting. With respect to alcohol consumption, we collected information on drinking status at the time of recruitment (never, former, or current), age when they started drinking, habitual drinking frequency (times/week), habitual drinking volume (mL/day) for each beverage, and age at quitting in case of former drinkers.

After filling out the self‐administered questionnaire, study subjects mailed them to the data management center or handed them to the attending physician at the time of regular consultation. Research technicians completed missing and corrected illogical data by communicating with the patients over the telephone or in a direct interview.

Each attending physician extracted the following clinical information about the patient from their medical records: laboratory data before treatment for HCV that achieved SVR (albumin [g/dL], total bilirubin [mg/dL], AST [IU/L], ALT [IU/L], platelet count [× 10^4^/μL], AFP [ng/mL]), HCV genotype, types of HCV treatment that achieved SVR and dates of starting and ending treatment, and history and date of HCC diagnosis. As an indicator of fibrosis before HCV treatment, the APRI was calculated as AST (IU/L)/upper limit of normal value for AST (i.e., 33 IU/L) divided by platelet count (×10^9^/L) and multiplied by 100.^10^, ^11^ We did not calculate the fibrosis‐4 (FIB4) index due to potential multicollinearity with age in statistical analyses.

For patients diagnosed with post‐SVR HCC, diagnostic imaging findings and histological findings at the time of HCC diagnosis were reviewed from patients' medical records in order to confirm the diagnosis of HCC. HCC surveillance is basically continued even after completion of SVR at a 3–12‐month interval with imaging, blood tests, and outpatient consultations.

### *Main exposures and covariates*

Each patient's smoking and drinking status at or after the time of SVR was considered the main exposure variable. We also prioritized age at the time of SVR, defined as age at the completion of treatment for HCV.

We estimated smoking status (never, former, or current) at the time of SVR for each patient using information on age at starting smoking, quitting smoking, and achieving SVR because we did not directly ask about “smoking status at the time of SVR” in the self‐administered questionnaire. For current smokers at the time of SVR, daily smoking amount after SVR (cigarettes/day), smoking period after SVR (years), and cumulative smoking amount after SVR (pack‐years) were classified into two categories based on the median value of current smokers. Smoking period after SVR was calculated by subtracting age at the time of SVR from age at recruitment, age at HCC diagnosis, or age at quitting smoking, as appropriate. Cumulative smoking amount after SVR was determined by multiplying daily smoking amount (packs) by smoking period after SVR (years), assuming that one pack contained 20 cigarettes.

Based on drinking status at the time of SVR, patients were classified as never, former, or current drinkers using the same procedure as smoking. For current drinkers at the time of SVR, habitual ethanol consumption after SVR (g/day), habitual drinking frequency after SVR (times/week), drinking period after SVR (years), and cumulative ethanol consumption after SVR (kg) were further classified into two categories based on the median value in current drinkers. Habitual ethanol consumption after SVR (g/day) was calculated according to the following formula: grams of ethanol = habitual drinking volume (mL) × percentage of ethanol in the drink × 0.8/100. Drinking period after SVR was calculated by subtracting age at the time of SVR from age at recruitment, age at HCC diagnosis, or age at quitting alcohol, as appropriate. Cumulative ethanol consumption after SVR was calculated according to the following formula: habitual ethanol consumption (g/day) × habitual drinking frequency (times/week) × 365/7 × drinking period after SVR (years).

For other covariates, including body mass index (BMI, kg/m^2^), medical history, and years since SVR, status at recruitment or HCC diagnosis was considered. BMI was classified into two categories based on the cutoff point for obesity in the Japanese population (<25/≥25). Laboratory data before treatment were classified into two levels using conventional cutoff values. APRI before treatment was classified into two categories with a threshold of 1.0 based on the findings of a meta‐analysis, in which a threshold of 1.0 showed 61% sensitivity and 64% specificity for severe fibrosis.^11^ For HCC patients whose APRI was not calculated due to missing laboratory data, APRI before treatment was considered to be <1 if their fibrosis stage according to histological findings at the time of HCC diagnosis was F1 according to the classification of Desmet *et al*.^12^ If the stage was F4, APRI before treatment was considered to be ≥1.

### *Statistical analysis*

The distribution of characteristics between HCC and non‐HCC patients was assessed using the Chi‐square test, Fisher's exact test, or Wilcoxon rank sum test, as appropriate. To examine the association with HCC, odds ratios (ORs) and 95% confidence intervals (CIs) were calculated using a logistic regression model. Variables showing associations with HCC in univariate analyses with *P* < 0.05 were considered potential confounders.

As laboratory data strongly correlated with each other, priority for inclusion in the multivariate model was given to APRI, which is commonly used in clinical studies. The type of HCV treatment that achieved SVR was not included in the multivariate model because a meta‐analysis reported no significant difference in the occurrence of HCC between IFN‐based and DAA treatments.^13^ Similarly, HCV genotype was not included because there are some reports indicating no significant difference in post‐SVR HCC morbidity between genotypes 1 and 2.^5^, ^7^, ^8^ Therefore, due to the small number of subjects, multivariate analysis included only gender and APRI before SVR as the most relevant confounders for HCC.

All analyses were performed using SAS version 9.4 software (SAS Institute, Cary, NC, USA).

## Results

**Figure 1:**
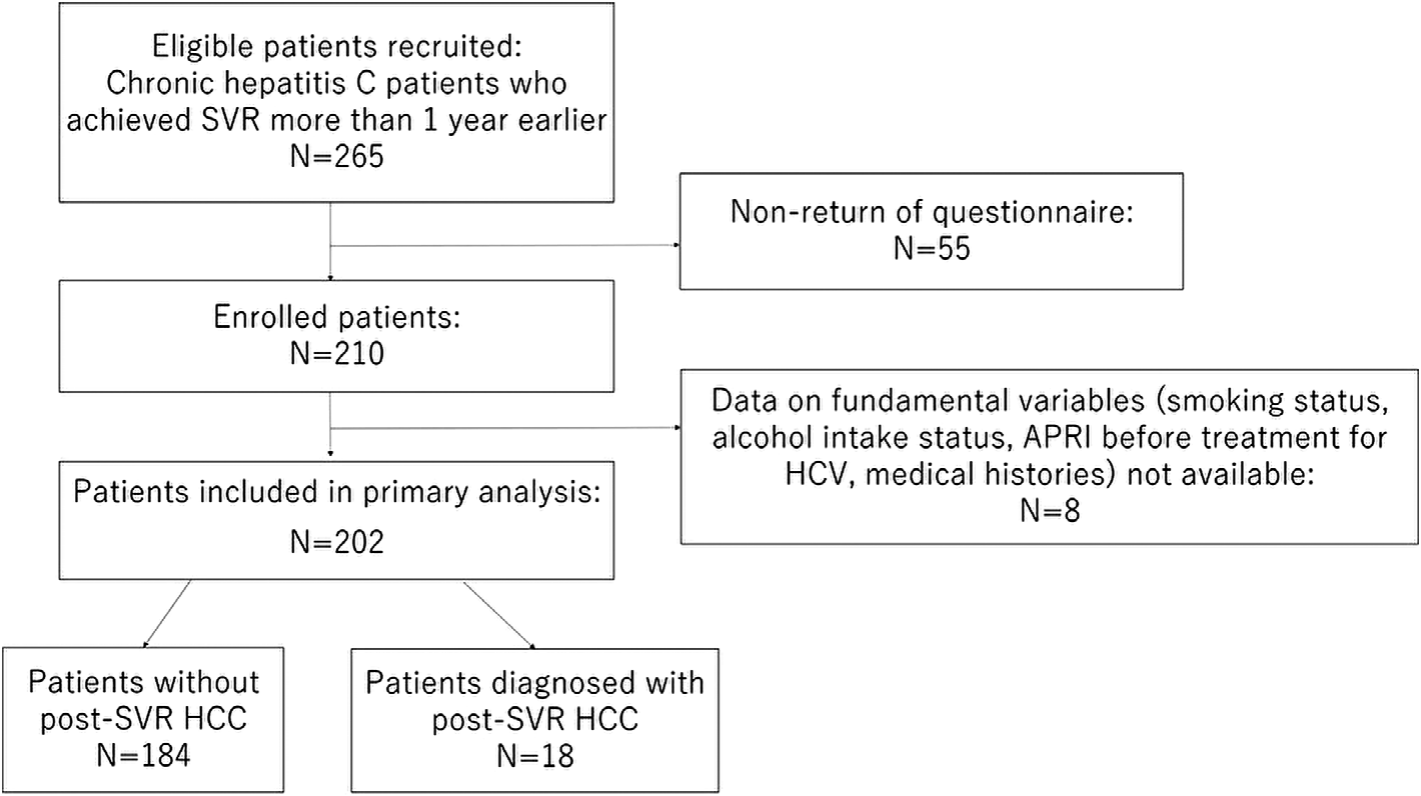
Flowchart of patient inclusion in the study. APRI, aspartate aminotransferase‐to‐platelet ratio index; HCC, hepatocellular carcinoma; HCV, hepatitis C virus; SVR, sustained virologic response.

Of the 265 eligible patients recruited, 210 patients returned the self‐administered questionnaire and were enrolled in the study (response rate: 79%). After excluding eight patients without information on fundamental variables (smoking habit, alcohol habit, APRI before treatment, medical histories), a total of 202 patients, including 18 HCC patients and 184 non‐HCC patients, were subjected to the primary analysis (Fig. 1).

Compared with non‐HCC patients, HCC patients were likely to be male, have histories of gastric or duodenal ulcers, achieved SVR with IFN, and had HCV genotype 2 infection (Table 1). There were no subjects with hyperlipidemia among the HCC patients. Besides, HCC patients had higher levels of AST, ALT, and APRI and lower platelet counts, indicating severe fibrosis before treatment. As for lifestyle habits at the time of SVR, HCC patients included more current smokers and former drinkers than non‐HCC patients.

**Table 1:** Characteristics of the study subjects (*N* = 202)

Characteristics	Non‐HCC (*N* = 184) *n* (%) or median (range)		HCC (*N* = 18) *n*(%) or median (range)		*P*‐value†
At the time of recruitment or HCC diagnosis					
Gender					
Male	73	(39)	15	(83)	< 0.01
Body mass index (kg/m^2^)					
≥ 25	43	(23)	5	(28)	0.77
Medical history (present)					
Diabetes	18	(10)	2	(11)	0.69
Hypertension	78	(43)	7	(39)	0.76
Missing	1		0		
Hyperlipidemia	53	(29)	0		< 0.01
Gastric or duodenal ulcer	33	(18)	10	(56)	< 0.01
Years since SVR	3	(1.0‐12.8)	3.5	(1.5‐21.0)	0.08
At the time of SVR‡					
Age	62.4	(21‐86)	61	(53‐75)	0.99
Smoking status					
Never	102	(55)	5	(28)	0.04
Former	49	(27)	6	(33)	
Current	33	(18)	7	(39)	
Alcohol drinking status					
Never	59	(32)	1	(6)	< 0.01
Former	30	(16)	9	(50)	
Current	95	(52)	8	(44)	
Before treatment that achieved SVR					
Type of HCV treatment					
IFN	127	(56)	18	(100)	< 0.01
IFN+DAA	50	(24)	0		
DAA	38	(21)	0		
HCV genotype					
1	127	(72)	4	(31)	< 0.01
2	50	(28)	9	(69)	
Missing	7		5		
Laboratory data					
Albumin (g/dl)					
< 3.5	10	(5)	1	(8)	0.54
Missing	1		5		
Total bilirubin (mg/dl)					
≥ 1.1	33	(18)	3	(25)	0.46
Missing	0		6		
Aspartate aminotransferase (IU/l)					
≥ 34	120	(65)	13	(100)	< 0.01
Missing	0		5		
Alanine aminotransferase (IU/l)					
≥ 46	88	(48)	13	(85)	< 0.01
Missing	0		5		
Platelet count (×10,000/μl)					
< 18	105	(57)	12	(92)	0.01
Missing	0		5		
Alpha‐fetoprotein (ng/ml)					
≥ 20	15	(10)	2	(18)	0.33
Missing	36		7		
APRI§					
≥ 1	66	(36)	13	(72)	< 0.01

APRI, aspartate aminotransferase‐to‐platelet ratio index; DAA, direct acting antiviral; HCC, hepatocellular carcinoma; HCV, hepatitis C virus; IFN, interferon; SVR, sustained virologic response.

† Chi‐square test, Fisher's exact test, or Wilcoxon rank sum test was used, as appropriate.

‡ At the time of SVR: At completing treatment that achieved SVR.

§ For five HCC patients whose laboratory data before treatment were not available, three patients were considered APRI <1 and two patients were APRI ≥ 1 based on histological findings at the time of HCC diagnosis.

After considering the effect of gender and APRI before treatment (<1 or ≥1), male gender (OR = 6.92, 95% CI = 1.91–25.12; *P* < 0.01), gastric or duodenal ulcer history (OR = 5.23, 95% CI = 1.74–15.76; *P* < 0.01), former drinker at the time of SVR (OR = 9.51, 95% CI = 1.08–83.90; *P* = 0.04), and APRI before treatment ≥1 (OR = 4.14, 95% CI = 1.37–12.46; *P* = 0.01) demonstrated a significantly increased OR for HCC (Table 2).

**Table 2:** Association between selected characteristics and post‐SVR HCC

Characteristics	HCC, n/N (%)	Crude OR (95% CI)	*P*‐value	Adjusted† OR (95% CI)	*P*‐value
Gender					
Male	15/88 (17%)	7.60 (2.13–27.19)	< 0.01	6.92 (1.91‐25.12)	< 0.01
Female	3/114 (3%)	1.00		1.00	
Gastric or duodenal ulcer history					
Absent	8/159 (5%)	1.00		1.00	
Present	10/43 (23%)	5.72 (2.10‐15.60)	< 0.01	5.23 (1.74‐15.76)	< 0.01
Smoking status at the time of SVR					
Never	5/107 (5%)	1.00		1.00	
Former	6/56 (11%)	2.50 (0.73‐8.59)	0.15	0.96 (0.24‐3.79)	0.97
Current	7/40 (18%)	4.33 (1.29‐14.55)	0.02	2.83 (0.72‐11.10)	0.14
		Trend *P*	0.02	Trend *P*	0.13
Alcohol drinking status at the time of SVR					
Never	1/60 (2%)	1.00		1.00	
Former	9/39 (23%)	17.69 (2.14‐146.18)	0.01	9.51 (1.08‐83.90)	0.04
Current	8/103 (8%)	4.97 (0.61‐40.70)	0.14	2.72 (0.31‐24.10)	0.38
		Trend *P*	0.37	Trend *P*	0.96
APRI before treatment					
< 1	6/124 (5%)	1.00		1.00	
≥ 1	12/78 (15%)	4.65 (1.59‐13.61)	< 0.01	4.14 (1.37‐12.46)	0.01

APRI, aspartate aminotransferase‐to‐platelet ratio index; CI, confidence interval; HCC, hepatocellular carcinoma; OR, odds ratio; SVR, sustained virologic response.

† Adjusted for gender, APRI before treatment (<1/≥1).

Regarding smoking habit (Table 3), current smokers at the time of SVR had a significantly increased OR for HCC in univariate analysis (OR = 4.33, 95% CI = 1.29–14.55; *P* = 0.02) but not in multivariate analysis. For daily smoking amount, smoking period, and cumulative smoking amount after SVR, no significant association was found in multivariate analysis.

**Table 3:** Associations between cigarette smoking and post‐SVR HCC

Characteristics	HCC, n/N (%)	Crude OR (95% CI)	*P*‐value	Adjusted† OR (95% CI)	*P*‐value
Smoking status at the time of SVR					
Never	5/107 (5%)	1.00		1.00	
Former	6/55 (11%)	2.50 (0.73–8.59)	0.15	0.96 (0.24–3.79)	0.95
Current	7/40 (18%)	4.33 (1.29–14.55)	0.02	2.83 (0.72–11.10)	0.14
		Trend *P*	0.02	Trend *P*	0.13
Daily smoking amount after SVR (cigarettes/day)					
Never	5/107 (5%)	1.00		1.00	
Former	6/55 (11%)	2.50 (0.73–8.59)	0.15	0.96 (0.24–3.82)	0.97
Current < 20	3/20 (15%)	3.60 (0.79–16.47)	0.09	2.64 (0.49–14.16)	0.25
≥ 20	3/18 (17%)	4.08 (0.88–18.85)	0.07	2.92 (0.52–16.35)	0.22
Missing	1/2	Trend *P*	0.03	Trend *P*	0.14
Smoking period after SVR (years)					
Never	5/107 (5%)	1.00		1.00	
Former	6/55 (11%)	2.50 (0.73–8.59)	0.15	0.96 (0.24–3.80)	0.95
Current < 2	3/20 (15%)	3.60 (0.79–16.47)	0.09	2.85 (0.51–15.75)	0.23
≥ 2	4/20 (20%)	5.10 (1.24–21.03)	0.02	2.82 (0.58–13.71)	0.20
		Trend *P*	0.01	Trend *P*	0.11
Cumulative smoking amount after SVR (pack‐years)					
Never	5/107 (5%)	1.00		1.00	
Former	6/55 (11%)	2.50 (0.73–8.59)	0.15	0.97 (0.25–3.83)	0.96
Current < 2	2/20 (10%)	2.27 (0.41–12.59)	0.35	2.01 (0.31–13.28)	0.47
≥ 2	4/18 (22%)	5.83 (1.40–24.32)	0.02	3.43 (0.70–16.78)	0.13
Missing	1/2	Trend *P*	0.02	Trend *P*	0.10

OR, odds ratio; CI, confidence interval; HCC, hepatocellular carcinoma; SVR, sustained virologic response; APRI, aspartate aminotransferase‐to‐platelet ratio index;

† Adjusted for gender, APRI before treatment (<1/≥1).

With respect to alcohol consumption (Table 4), both univariate and multivariate analysis showed that former drinkers at the time of SVR had a significant association with an increased risk of HCC (adjusted OR = 9.51, 95% CI = 1.08–83.90; *P* = 0.04). In contrast, current drinkers at the time of SVR had no significant association with HCC in both univariate and multivariate analyses compared to never drinkers. Furthermore, there was no significant association even if current drinkers at the time of SVR were categorized as light drinkers or heavy drinkers according to habitual ethanol consumption, habitual drinking frequency, drinking period, and cumulative ethanol consumption after SVR.

**Table 4:** Associations between alcohol drinking and post‐SVR HCC

Characteristics	HCC, n/N (%)	Crude OR (95% CI)	*P*‐value	Adjusted† OR (95% CI)	*P*‐value
Alcohol drinking status at the time of SVR					
Never	1/60 (2%)	1.00		1.00	
Former	9/39 (23%)	17.69 (2.14–146.18)	0.01	9.51 (1.08–83.90)	0.04
Current	8/103 (8%)	4.97 (0.61–40.70)	0.14	2.72 (0.31–24.10)	0.37
		Trend *P*	0.37	Trend *P*	0.96
Habitual ethanol consumption after SVR (g/day)					
Never	1/60 (2%)	1.00		1.00	
Former	9/39 (23%)	17.69 (2.14–146.17)	0.01	9.51 (1.08–83.95)	0.04
Current < 29.6	4/51 (8%)	5.02 (0.54–46.40)	0.16	3.48 (0.35–34.85)	0.29
≥ 29.6	4/50 (8%)	5.13 (0.55–47.43)	0.15	2.26 (0.22–23.06)	0.49
Missing	0/2	Trend *P*	0.55	Trend *P*	0.61
Habitual drinking frequency after SVR (times/week)					
Never	1/60 (2%)	1.00		1.00	
Former	9/39 (23%)	17.69 (2.14–146.17)	0.01	9.43 (1.07–83.42)	0.04
Current < 3.5	4/50 (8%)	5.13 (0.55–47.43)	0.15	3.59 (0.36–35.83)	0.28
≥ 3.5	4/51 (8%)	5.02 (0.54–46.40)	0.16	2.26 (0.22–23.06)	0.49
Missing	0/2	Trend *P*	0.57	Trend *P*	0.62
Drinking period after SVR (years)					
Never	1/60 (2%)	1.00		1.00	
Former	9/39 (23%)	17.69 (2.14–146.18)	0.01	9.51 (1.08–83.88)	0.04
Current < 3.8	4/51 (8%)	5.02 (0.54–46.40)	0.16	2.58 (0.26–26.01)	0.42
≥ 3.8	4/52 (8%)	4.91 (0.53–45.42)	0.16	2.86 (0.29–28.76)	0.37
		Trend *P*	0.59	Trend *P*	0.79
Cumulative ethanol consumption after SVR (kg)					
Never	1/60 (2%)	1.00		1.00	
Former	9/39 (23%)	17.69 (2.14–146.17)	0.01	9.52 (1.08–84.09)	0.04
Current < 7.8	4/50 (8%)	5.13 (0.55–47.43)	0.15	3.18 (0.32–31.82)	0.32
≥ 7.8	4/50 (8%)	5.13 (0.55–47.43)	0.15	2.58 (0.25–26.85)	0.43
Missing	0/3	Trend *P*	0.54	Trend *P*	0.73

OR, odds ratio; CI, confidence interval; HCC, hepatocellular carcinoma; SVR, sustained virologic response; APRI, aspartate aminotransferase‐to‐platelet ratio index.

† Adjusted for gender, APRI before treatment (<1/≥1).

When we limited our subjects to patients treated with IFN, the results did not change markedly from the findings among all subjects (Tables S1–S3).

Tables 5 and 6 show the results of the analysis stratified according to APRI before treatment (≥1/<1). In patients with APRI ≥ 1 before treatment, OR for HCC was significantly increased in patients with a history of gastric or duodenal ulcer (OR = 9.30, 95% CI = 2.39–36.18; *P* < 0.01), and current smokers at the time of SVR had a slightly increased OR for HCC compared to never smokers (OR = 5.61, 95% CI = 0.97–32.63; *P* = 0.05) (Table 5). In addition, compared to never smokers, current smokers who smoked fewer than 20 cigarettes per day (OR = 8.58, 95% CI = 1.09–67.81; *P* = 0.04) or smoked for at least 2 years after SVR (OR = 7.93, 95% CI = 1.16–54.18; *P* = 0.03) had a significantly increased OR for HCC (Table 6).

**Table 5:** Additional analyses regarding association between selected characteristics and post‐SVR HCC stratified by APRI before treatment

Characteristics	APRI before treatment ≥ 1 (*N* = 79)			APRI before treatment < 1 (*N* = 123)		
	HCC, n/N (%)	Adjusted† OR (95% CI)	*P*‐value	HCC, n/N (%)	Adjusted† OR (95% CI)	*P*‐value
Gastric or duodenal ulcer history						
Absent	5/62 (8%)	1.00		3/97 (3%)	1.00	
Present	8/17 (47%)	9.30 (2.39–36.18)	< 0.01	2/26 (8%)	1.92 (0.17–21.40)	0.60
Smoking habit at the time of SVR						
Never	3/39 (8%)	1.00		2/68 (3%)	1.00	
Former	5/28 (18%)	1.65 (0.31–8.78)	0.56	1/27 (4%)	0.20 (0.01–5.75)	0.35
Current	5/12 (42%)	5.61 (0.97–32.63)	0.05	2/28 (7%)	0.95 (0.07–13.51)	0.97
		Trend *P*	0.05		Trend *P*	0.96
Alcohol drinking habit at the time of SVR						
Never	1/22 (5%)	1.00		0/38 (0%)	1.00	
Former	7/22 (32%)	14.52 (0.61–344.22)	0.09	2/17 (12%)	NA	
Current	5/35 (14%)	1.05 (0.06–18.80)	0.98	3/68 (4%)	NA	
		Trend *P*	0.38			

APRI, aspartate aminotransferase‐to‐platelet ratio index; CI, confidence interval; HCC, hepatocellular carcinoma; NA, not available; OR, odds ratio; SVR, sustained virologic response.

† Adjusted for gender.

**Table 6:** Additional analyses regarding associations between cigarette smoking and post‐SVR HCC stratified by APRI before treatment

Characteristics	APRI before treatment ≥ 1 (*N* = 79)			APRI before treatment < 1 (*N* = 123)		
	HCC, n/N (%)	Adjusted† OR (95% CI)	*P*‐value	HCC, n/N (%)	Adjusted† OR (95% CI)	*P*‐value
Smoking status at the time of SVR						
Never	3/39 (8%)	1.00		2/68 (3%)	1.00	
Former	5/28 (18%)	1.65 (0.31–8.78)	0.56	1/27 (4%)	0.38 (0.01–5.75)	0.45
Current	5/12 (42%)	5.61 (0.97–32.63)	0.05	2/28 (7%)	1.00 (0.12–8.21)	1.00
		Trend *P*	0.05		Trend *P*	1.00
Daily smoking amount after SVR (cigarettes/day)						
Never	3/39 (8%)	1.00		2/68 (3%)	1.00	
Former	5/28 (18%)	1.62 (0.30–8.67)	0.57	1/27 (4%)	0.38 (0.03–4.69)	0.45
Current < 20	3/6 (50%)	8.58 (1.09–67.81)	0.04	0/14 (0%)	NA	0.96
≥ 20	1/4 (25%)	2.52 (0.18–35.98)	0.50	2/14 (14%)	1.86 (0.21–16.18)	0.58
Missing	1/2	Trend *P*	0.12		Trend *P*	0.64
Smoking period after SVR (years)						
Never	3/39 (8%)	1.00		2/68 (3%)	1.00	
Former	5/28 (18%)	1.64 (0.31–8.73)	0.56	1/27 (4%)	0.38 (0.03–4.69)	0.45
Current < 2	1/4 (25%)	2.54 (0.18–36.26)	0.49	2/16 (13%)	1.86 (0.21–16.18)	0.58
≥ 2	4/8 (50%)	7.93 (1.16–54.18)	0.03	0/12 (0%)	NA	
		Trend *P*	0.03		Trend *P*	0.77
Cumulative smoking amount after SVR (pack‐years)						
Never	3/39 (8%)	1.00		2/68 (3%)	1.00	
Former	5/28 (18%)	1.66 (0.31–8.85)	0.72	1/27 (4%)	0.38 (0.03–4.69)	0.45
Current < 2	1/3 (33%)	4.21 (0.26–66.88)	0.47	1/17 (6%)	0.81 (0.06–10.48)	0.87
≥ 2	3/7 (43%)	6.14 (0.83–45.47)	0.09	1/11 (9%)	1.30 (0.10–17.73)	0.84
Missing	1/2	Trend *P*	0.06		Trend *P*	0.86

OR, odds ratio; CI, confidence interval; HCC, hepatocellular carcinoma; SVR, sustained virologic response; APRI, aspartate aminotransferase‐to‐platelet ratio index; NA, not available;

† Adjusted for gender.

## Discussion

In the present study, we confirmed that male gender and severe fibrosis before treatment (APRI ≥ 1) were risk factors for post‐SVR HCC, as previously established. In addition, former drinkers at the time of SVR and history of gastric or duodenal ulcer were shown to increase the risk of post‐SVR HCC. We also found that, among patients with severe fibrosis before SVR, current smoking at the time of SVR could be a risk factor for HCC.

### *Smoking*

A systematic review focusing on the general population reported that smoking was an independent risk factor for HCC.^14^ In addition, patients with chronic hepatitis C had a higher risk of developing HCC due to smoking, and a meta‐analysis showed a multiplicative interaction between HCV infection and smoking.^9^ Some chemicals present in tobacco smoke, including 4‐aminobiphenyl and polycyclic aromatic hydrocarbons, might be metabolized to carcinogens in the liver.^15^, ^16^, ^17^ In contrast, a cohort study that followed chronic hepatitis C patients with compensated cirrhosis for 5 years did not show an increased risk of HCC due to smoking.^18^ In the present study, multivariate analysis showed that continuation of smoking after SVR did not significantly increase the risk of HCC. However, an analysis stratified according to APRI before treatment suggested that, in patients with severe hepatic fibrosis before SVR, continuation of smoking increased the risk of HCC. In addition, 6 of 18 HCC cases developed HCC more than 5 years after SVR (data not shown). This suggests that long‐term follow up of high‐risk populations, such as men with liver fibrosis, might reveal an increased risk of HCC due to smoking.

### *Alcohol consumption*

Heavy alcohol consumption is a well‐known risk factor for HCC.^19^, ^20^, ^21^ There are several reports showing that, in patients with chronic hepatitis C, fibrosis progressed with moderate alcohol consumption due to the synergistic effect of HCV infection and alcohol consumption.^21^, ^22^ Although the mechanism by which alcohol consumption causes HCC has not been fully elucidated, most cases developed through the processes of liver steatosis, liver fibrosis, and cirrhosis.^23^ In the present study, drinking after achieving SVR did not increase the risk of developing post‐SVR HCC. Conversely, former drinkers showed a significant increase in the risk of HCC. One plausible explanation for these results was that heavy drinkers might have quit drinking before SVR due to progression of liver disease. As a result, only light‐to‐moderate drinkers were able to continue drinking. Another possibility could be that drinking habits before achieving SVR have a greater effect on HCC risk than drinking habits after SVR due to the interaction between HCV infection and alcohol consumption. In addition, in the cohort study of patients with compensated cirrhosis described above, mild to moderate alcohol consumption increased the 5‐year cumulative incidence of HCC even in patients who achieved SVR.^18^ Thus, it is recommendable for patients with severe liver fibrosis to stop drinking even after achieving SVR.

### *Gastric or duodenal ulcer history*

Unexpectedly, we found that patients with histories of gastric or duodenal ulcers had significantly increased OR for post‐SVR HCC. However, gastric or duodenal ulcer history might be a surrogate for other factors, such as heavy smoking or alcohol consumption before achieving SVR, *Helicobacter pylori* (*H. pylori*) infection, medications including proton pump inhibitors (PPI) or histamine 2 receptor blockers, and iron overload due to blood transfusion. Although we simultaneously adjusted for a smoking or drinking habit at the time of SVR in calculating the OR of gastric or duodenal ulcer history, residual confounding could not be denied. As for *H. pylori* infection, a previous meta‐analysis reported a positive association between *H. pylori* and the risk of HCC.^24^ However, direct evidence of the tumorigenic effect of *H. pylori* on the liver has not been obtained.^25^, ^26^ With regard to the effect of medication use, a previous report stated that even the use of PPIs was associated with increased risk of HCC in chronic hepatitis C patients.^27^ As information on the prevalence of *H. pylori* antibodies in serum and history of medication usage were not available in this study, the possible causal relationship did not go beyond speculation. Iron deposition in the liver by blood transfusion might also have affected the risk of HCC in our study subjects because the proportion of self‐reported transfusion histories was higher in the HCC group than the non‐HCC group (53% *vs* 30%, *P* = 0.05).

### *Strength*

A strength of this study was the ability to consider multiple confounders by combining the information from medical charts and self‐administered questionnaires. In many previous studies on risk factors for post‐SVR HCC, laboratory data and histological findings were collected from medical records or registries, while the relationship between post‐SVR HCC and lifestyle factors, such as smoking and drinking habits, was rarely evaluated, although these might change after SVR. In this study, we comprehensively investigated the association between smoking and drinking habits and post‐SVR HCC by obtaining detailed information on patient lifestyle factors from the questionnaire.

### *Limitation*

This study has certain limitations. First, it lacked statistical power due to the small number of study subjects. Second, because it was a cross‐sectional study rather than a cohort study, the time‐dependent occurrence of HCC could not be evaluated. Third, regarding the characteristics of participants (Table 1), more HCC patients had HCV genotype 2, and all HCC patients achieved SVR with IFN, which was probably due to selection bias. In addition, the study was also limited to those who survived until recruitment and did not include patients who had died of HCC or cirrhosis. Fourth, because information about drinking and smoking in this study was based on patient self‐administered questionnaires, there might have been misclassification during information collection.

## Conclusion

Continued smoking might be a risk factor for HCC after SVR, especially in chronic hepatitis C patients with severe fibrosis. Therefore, it is important to advise patients to quit smoking even after achieving SVR. Furthermore, a history of gastric or duodenal ulcers might be a risk factor for developing HCC, which needs further investigation. Future prospective studies in patients who achieve SVR with IFN‐free treatment are required to validate the results of our study.

## Supporting information


**Appendix** **S1.** Supporting Information
