# Supplementary material for: Risk factors for hepatocellular carcinoma in treated chronic hepatitis C patients–Relationship to smoking and alcohol
Source: JGH Open. 2020 Apr 16;4(5):867–75. doi: 10.1002/jgh3.12331 (PMC7578324; doi:10.1002/jgh3.12331)
Supplement: Supplementary file 1 [file JGH3-4-867-s001.docx]

Supplementary table S1. Association between selected characteristics and post-SVR HCC in patients treated with interferon (N=164)

Characteristics	HCC prevalence n/N (%)	Crude OR (95%CI)	*P*-value	Adjusted† OR (95%CI)
Gender				
Male	15 / 73 (21%)	7.59 (2.10 - 27.37)	< 0.01	6.79 (1.83 - 25.14
Female	3 / 91 (3%)	1.00		1.00
Gastric or duodenal ulcer history				
Absent	8 / 125 (6%)	1.00		1.00
Present	10 / 39 (26%)	5.04 (1.83 - 13.91)	< 0.01	4.26 (1.36 - 13.35
Smoking status at the time of SVR				
Never	5 / 82 (6%)	1.00		1.00
Former	6 / 47 (13%)	2.25 (0.65 - 7.83)	0.20	0.84 (0.21 - 3.45
Current	7 / 35 (20%)	3.85 (1.13 - 13.13)	0.03	2.33 (0.58 - 9.43
		Trend P	0.03	Trend P
Alcohol drinking status at the time of SVR				
Never	1 / 43 (2%)	1.00		1.00
Former	9 / 33 (27%)	15.75 (1.88 - 131.96)	0.01	9.45 (1.03 - 86.70
Current	8 / 88 (9%)	4.20 (0.51 - 34.70)	0.18	2.53 (0.28 - 22.74
		Trend P	0.55	Trend P
APRI before treatment				
< 1	5 / 106 (5%)	1.00		1.00
≥ 1	13 / 58 (22%)	5.84 (1.96 - 17.35)	< 0.01	5.19 (1.69 - 15.96

CONFIDE

Supplementary table S2. Associations between cigarette smoking and post-SVR HCC in patients treated with interferon (N

Characteristics		HCC prevalence n/N (%)	Crude OR (95%CI)	*P*-value	Adjusted† OR (
Smoking status at the time of SVR					
Never		5 / 82 (6%)	1.00		1.00
Former		6 / 47 (13%)	2.25 (0.65 - 7.83)	0.20	0.84 (0.21 -
Current		7 / 35 (20%)	3.85 (1.13 - 13.13)	0.03	2.33 (0.58 -
			Trend *P*	0.03	
Daily smoking amount after SVR (cigarettes/day)					
Never		5 / 82 (6%)	1.00		1.00
Former		6 / 47 (13%)	2.25 (0.65 - 7.83)	0.20	0.84 (0.20 -
Current	< 20	3 / 17 (18%)	3.30 (0.71 - 15.40)	0.13	2.09 (0.36 -
	≥ 20	3 / 16 (19%)	3.55 (0.76 - 16.70)	0.11	2.71 (0.46 -
	Missing	1 / 2	Trend *P*	0.06	
Smoking period after SVR (years)					
Never		5 / 82 (6%)	1.00		1.00
Former		6 / 47 (13%)	2.25 (0.65 - 7.83)	0.20	0.83 (0.20 -
Current	< 2	3 / 15 (20%)	3.85 (0.81 - 18.23)	0.09	3.23 (0.54 -
	≥ 2	4 / 20 (20%)	3.85 (0.93 - 15.94)	0.06	1.88 (0.37 -
			Trend *P*	0.03	
Cumulative smoking amount after SVR (pack-years)					
Never		5 / 82 (6%)	1.00		1.00
Former		6 / 47 (13%)	2.25 (0.65 - 7.83)	0.20	0.84 (0.20 -
Current	< 2	2 / 15 (13%)	2.37 (0.42 - 13.52)	0.33	2.43 (0.35 -
	≥ 2	4 / 18 (22%)	4.40 (1.05 - 18.44)	0.04	2.33 (0.46 -
	Missing	1 / 2	Trend *P*	0.04	

CONFID

Supplementary table S3. Associations between alcohol drinking and post-SVR HCC in patients treated with interferon (N=164)

Characteristics		HCC prevalence n/N (%)	Crude OR (95%CI)	*P*-value	Adjusted† OR (95%CI)
Alcohol drinking status at the time of SVR					
Never		1 / 43 (2%)	1.00		1.00
Former		9 / 33 (27%)	15.75 (1.88 - 131.96)	0.01	9.45 (1.03 - 86.[illegible]
Current		8 / 88 (9%)	4.20 (0.51 - 34.70)	0.18	2.53 (0.28 - 22.[illegible]
			Trend *P*	0.55	Trend P
Habitual ethanol consumption after SVR (g/day)					
Never		1 / 43 (2%)	1.00		1.00
Former		9 / 33 (27%)	15.75 (1.88 - 131.95)	0.01	9.45 (1.03 - 86.[illegible]
Current	< 29.6	4 / 46 (9%)	4.00 (0.43 - 37.29)	0.22	3.09 (0.30 - 31.[illegible]
	≥ 29.6	4 / 41 (10%)	4.54 (0.49 - 42.44)	0.18	2.15 (0.21 - 22.[illegible]
	Missing	0 / 1	Trend *P*	0.72	Trend *P*
Habitual drinking frequency after SVR (times/week)					
Never		1 / 43 (2%)	1.00		1.00
Former		9 / 33 (27%)	15.75 (1.88 - 131.96)	0.01	9.46 (1.02 - 87.[illegible]
Current	< 3.5	4 / 43 (9%)	4.31 (0.46 - 40.22)	0.20	3.71 (0.36 - 38.[illegible]
	≥ 3.5	4 / 43 (9%)	4.31 (0.46 - 40.22)	0.20	2.02 (0.20 - 20.[illegible]
	Missing	0 / 2	Trend *P*	0.75	Trend *P*
Drinking period after SVR (years)					
Never		1 / 43 (2%)	1.00		1.00
Former		9 / 33 (27%)	15.75 (1.88 - 131.96)	0.01	9.45 (1.03 - 86.[illegible]
Current	< 3.8	4 / 36 (11%)	5.25 (0.56 - 49.25)	0.15	3.35 (0.32 - 34.[illegible]
	≥ 3.8	4 / 52 (8%)	3.50 (0.38 - 32.54)	0.27	2.03 (0.20 - 20.[illegible]
			Trend *P*	0.94	Trend *P*
Cumulatve ethanol consumption after SVR (kg)					
Never		1 / 43 (2%)	1.00		1.00
Former		9 / 33 (27%)	15.75 (1.88 - 131.96)	0.01	9.45 (1.02 - 87.[illegible]
Current	< 7.8	4 / 41 (10%)	4.54 (0.49 - 42.44)	0.18	3.75 (0.36 - 38.[illegible]
	≥ 7.8	4 / 45 (9%)	4.10 (0.44 - 38.21)	0.22	2.01 (0.19 - 20.[illegible]
	Missing	0 / 2	Trend *P*	0.79	Trend *P*

CONF
